# Preliminary quantitative analysis of renal sinus fat dysfunction in T2DM patients using MRI fat fraction and R2* mapping

**DOI:** 10.3389/fendo.2025.1486839

**Published:** 2025-02-19

**Authors:** Yifan Dong, Qinhe Zhang, Xun Wang, Yuhui Liu, Qi An, Ziting Zhang, Lifang Hu, Liangjie Lin, Ailian Liu

**Affiliations:** ^1^ Department of Radiology, the First Affiliated Hospital of Dalian Medical University, Dalian, China; ^2^ Department of Medical Imaging, Dalian Medical University, Dalian, China; ^3^ Clinical & Technical Solutions, Philips Healthcare, Beijing, China

**Keywords:** renal sinus fat, fat dysfunction, type 2 diabetes mellitus, magnetic resonance imaging, radiomarker

## Abstract

**Purpose:**

To quantitatively analyze renal sinus fat (RSF) dysfunction in type 2 diabetes mellitus (T2DM) patients using magnetic resonance imaging (MRI) fat fraction (FF) and R2* mapping.

**Methods:**

The inpatients who underwent 1.5 T MRI examination (including MRI FF and R2* mapping) of the abdomen from January 2017 to December 2023 were enrolled. The RSF volume, FF and R2* of the right and left kidneys and the mean values were measured. Associations between mean FF and R2* value of RSF and T2DM were assessed with logistic regression. Receiver operating characteristic (ROC) curve was applied to calculate area under the curve (AUC) for the parameters to identify T2DM patients. Partial correlation coefficients after controlling for age, sex, and BMI were computed to analyze the correlations among the mean RSF volume, FF and R2*.

**Results:**

A total of 186 participants were finally enrolled in this study including 38 patients in T2DM group and 148 patients in non-T2DM group. Univariate logistic regression analyses showed the significant correlations of mean RSF FF (OR: 1.111, 95%CI: 1.054 - 1.171), P < 0.001) and R2* (OR: 1.120, 95%CI: 1.013 - 1.237), P = 0.027) with T2DM. Multivariate analysis showed that mean RSF FF (OR: 1.231, 95% CI: 1.098 - 1.380) is independently associated with T2DM after adjusting for age, sex and BMI. The AUC of mean RSF FF was 0.701 (0.630 - 0.766) with the sensitivity and specificity of 57.89% and 75.68%, respectively, when using 34.40% as the cut-off value. The AUC of mean RSF R2* was 0.616 (0.542 - 0.686) with the sensitivity and specificity of 68.42% and 58.11%, respectively, when using 21.97 Hz as the cut-off value. Furthermore, mean RSF FF presents significantly higher diagnostic efficacy for T2DM than R2* (P < 0.05). And combining mean RSF FF and R2* improved the diagnostic performance (AUC = 0.729).

**Conclusion:**

Mean RSF FF and R2* were significantly associated with T2DM, and mean RSF FF was the independent risk factor of T2DM. This finding indicates the hypertrophy of adipocytes and excessive iron deposition and hypoxia in RSF, which may represent dysfunction of RSF for T2DM.

## 1 Introduction

In recent years, the prevalence of type 2 diabetes mellitus (T2DM) has reached an alarming level. In 2021, as the tenth leading cause of death in the world, diabetes caused a total of 79.2 million disability-adjusted life years globally with approximately 529 million people living with the disease worldwide and T2DM accounted for more than 96% as the main driver of diabetes prevalence. By 2050, the number of people with diabetes worldwide will reach 1.31 billion, representing a substantial burden to healthcare systems (1, 2).

The renal sinus (RS) is located in the middle of the kidney containing the renal hilum and is bordered by renal parenchyma laterally. Renal sinus fat (RSF) refers to the ectopic perivascular fat depot around renal hilum, which is in close contact with renal vasculature, lymphatic vessel, renal pelvis and calyces. Moreover, it is also a component of visceral adipose tissue. RSF has distinct characteristics compared to other fat depots and impacts human metabolism through various mechanisms including endocrine, inflammation, and immune regulation. Additionally, excessive RSF can lead to compression of renal blood vessels and kidneys (3, 4).

Recent studies have shown that T2DM is associated with RSF. Excessive RSF accumulation has been observed to be more prevalent in patients with T2DM compared to healthy controls, even after adjusting for age, sex, and ethnicity (5). Adipocyte hyperplasia and excessive hypertrophy of existing adipocytes may contribute to a larger volume of RSF in T2DM patients (3). Specifically, excessively large, hypertrophied adipocytes become inadequately vascularized and hypoxic, resulting in cell stress, apoptosis, immune cell infiltration, iron excess, and dysregulated release of cytokines and adipokines ultimately leading to RSF dysfunction, related to insulin resistance (IR), a significant risk factor for the development of T2DM (6–9).

Compared to RSF volume, there is limited knowledge about the quality of RSF in T2DM patients, which can be associated with adipose tissue dysfunction (10). Magnetic resonance imaging (MRI) 3D multi-echo fat water separation technique is currently the optimal method for noninvasive and precise measurement of fat content in human tissues (11). Additionally, it is a viable approach for obtaining R2* (apparent spin-spin relaxation rate) values to estimate oxygenation status in specific tissues by considering the impact of paramagnetic substances on the T2* (apparent spin-spin relaxation time) signal (12–16).

In previous research, we measured the volume and fat fraction (FF) of RSF in normal Chinese subjects based on FF mapping and explored the correlations between them and biometric parameters (e.g., age and gender) (17). Nevertheless, the precise nature of RSF as a form of ectopic fat and its physiological alterations in patients with T2DM remain poorly understood. To the best of our knowledge, to date, in contrast to studies on ectopic fat in other organs or body regions such as the pancreas and liver, visceral and subcutaneous adipose tissues, there was a lack of studies about RSF dysfunction in T2DM patients by evaluating the variation of RSF FF and R2* (18, 19).

We hypothesized that the RSF FF in patients with T2DM could reflect hypertrophy of adipose tissue in the renal sinus, and R2* could reflect hypoxia in RSF. Therefore, in this study, we aimed to quantitative analyze of RSF dysfunction in T2DM patients using MRI FF and R2* mapping.

## 2 Methods

### 2.1 Study design and participants

This retrospective study, conducted at a single center, collected the data of hospitalized patients who underwent 1.5 T MRI examination of the abdomen from January 2017 and December 2023, including MRI FF and R2* mapping sequences. The images of the enrolled patients all incorporated clear and intact RS. Exclusion criteria were as follows: ①Insufficient clinical information; ②Age < 18 years; ③Weight changes by more than 5% within one month; ④A history of alcohol addiction (alcohol intake ≥ 210 g/week for men and 140 g/week for women in the past 10 years); ⑤Pregnancy; ⑥History of radiotherapy, chemotherapy, immunosuppressive therapy, antiviral therapy, and endocrine therapy; ⑦History of renal surgery, hydronephrosis, renal sinus mass, renal dysfunction or renal malformation. Finally, a total of 186 subjects (82 men and 104 women) were covered in the analysis.

Diagnostic criteria for T2DM include fasting plasma glucose (FPG) ≥ 7.0 mmol/L or receiving oral hypoglycemic medication or insulin treatment. This study was approved by our hospital ethics committee. And the waiver of informed consent was approved for the collection of data.

### 2.2 MRI examinations

In this study, MRI examination was performed on a 1.5 T scanner (GE Medical Systems, Inc., Waukesha, WI, USA), with an eight-channel phased-array body coil. All patients fasted for 4-6 hours and received pre-scanning instructions about how to exhale and hold their breath for more than 20 seconds. The subjects were placed in the supine position during the examination. A three-plane localization imaging gradient-echo sequence was performed at the beginning of the acquisition. We obtained the MRI FF and R2* mapping using iterative decomposition of water and fat with echo asymmetry and least square estimation-iron quantification (IDEAL-IQ), with the scanning parameters as follows: TR = 13.4 ms, TE = 4.8 ms, FOV = 36 × 36 cm^2^, matrix = 256 × 160, NEX = 1, bandwidth = 125kHz, layer thickness = 10 mm, layer spacing = 0, layer number = 24, breath holding for less than 24s, and flip angle = 5^◦^. Multiple acquired echo signals were collected during a single breath-hold, and the water-phase, fat-phase, in-phase, out-phase, R2* and fat fraction mapping were generated after reconstruction.

### 2.3 Measurement of renal sinus fat

**Figure 1:**
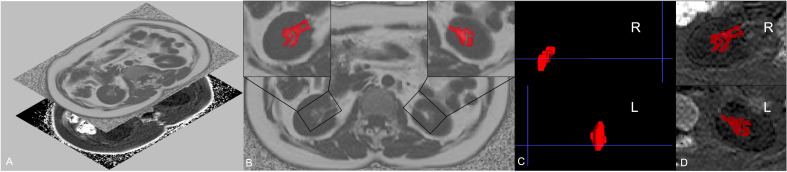
Regions of interest (ROIs) of left and right renal sinus fat. Fat fraction and R2* mapping can be automatically reconstructed after the scanning of IDEAL-IQ sequence, without the need for manual alignment **(A)**. Considering that renal sinus fat shows high signal on fat fraction mapping, the segmentation of renal sinus fat of both sides was firstly delineated on fat fraction maps **(B)**. The 3D label segmentations **(C)** were then extracted and matched on the R2* mapping **(D)**.

RSF was characterized as an ectopic perivascular fat depot around the renal hilum, which is close to the renal vasculature, lymphatic vessels, renal pelvis and calyces (5, 20). All slices of upper-middle abdominal MRI FF mapping were selected for RSF analysis (about 20 - 24 slices, slice thickness 10 mm) using the open-source software ITK-SNAP (v.3.6.0, http://www.itksnap.org/), with manual segmentation of RSF performed by the radiologist with 10 years of experience in abdominal imaging MR diagnosis. Based on the anatomical structure of the kidney, RSF of both kidneys was identified on MRI FF mapping by a straight-line tangent to the parenchymal edges adjacent to the renal hilum in axial slices. Subsequently, the adipose tissue in the bilateral RS was manually segmented, with the structures of renal lymphatics, veins, and ureters being excluded. After the scanning of IDEAL-IQ sequence, fat fraction and R2* mappings can be automatically reconstructed, without the need for manual alignment (**Figure 1A**). Due to the adipose tissue has high signal intensity on FF mapping, the RSF labeling process initiated from the upper pole of the kidney, marking the high signal tissues in the renal sinus area, and continued downwards until reaching the lower pole of the kidney on FF mapping (**Figures 1B, C**). And the labels we made on FF mapping were automatically put on the same region on R2* mapping and measured (**Figure 1D**).

Finally, the RSF volume, FF and R2* of the right and left kidneys and the mean values of both sides were automatically calculated using homemade software based on MATLAB (MATLAB R2018a). Specifically, mean RSF FF and mean RSF R2* are weighted averages according to the proportion of the volume of the right and left kidney.

### 2.4 Inter- and intra-observer variability

The intra-observer variability was assessed through repeated MRI-acquired fat measurements of 30 randomly selected patients taken by the same observer (with 10 years of experience in abdominal imaging MR diagnosis), with a minimum interval of four weeks between the two measurements. Inter-observer variability was determined by having a second independent observer (with 7 years of experience in abdominal imaging MR diagnosis) perform repeated measurements on the same patients using the identical method. Two radiologists involved in this study were blinded to the grouping.

### 2.5 Statistical analysis

All data were analyzed by GraphPad Prism (Version 8.4.0, GraphPad software, LLC) and MedCalc (Version 22.009, MedCalc Software bvba, Ostend, Belgium). The intraclass correlation coefficient (ICC) was used to assess the reliability of MRI-acquired fat measurements. The ICC was used to assess the consistency between the two observers: ICC < 0.4 indicated poor consistency; 0.4 ≤ ICC ≤ 0.75 indicated moderate consistency; ICC > 0.75 indicated good consistency. The Kolmogorov - Smirnov test was used to verify the normality assumption of continuous variables.

Normally distributed data were presented by mean ± standard deviation, and non-normally distributed data were presented by median with interquartile range (25th percentile, 75th percentile). Categorical variables were expressed as frequencies and percentages.

Comparisons between T2DM and non-T2DM group were analyzed using the two-sided independent sample t-test for normally distributed continuous variables, the non-parametric Mann - Whitney U-test for non-normally distributed continuous variables, and the Chi-square test for categorical variables.

Associations of mean RSF FF and R2* values to the presence of T2DM were assessed with univariable and multivariable logistic regression. Receiver operating characteristic (ROC) curve was applied to calculate area under the curve (AUC) for the parameters of RSF to identify T2DM patients. Additionally, the optimal cut-off value, sensitivity, and specificity were determined by calculating the Youden index. Delong test was used to compare the AUC values.

To analyze the correlations among the mean volume, FF and R2* value of RSF, partial correlation coefficients (r) after controlling for age, sex, and BMI were computed. Correlation coefficients were clarified as follows: weak, 0 - 0.4; moderate, 0.4 - 0.7; and strong, 0.7 - 1.0.

Calculated two-tailed P < 0.05 was considered statistically significant.

## 3 Results

### 3.1 Study subject characteristics

A total of 186 participants were finally enrolled in this study including 38 patients in the T2DM group (18 men and 20 women) and 148 patients in the non-T2DM group (64 men and 84 women). The average age of patients in the T2DM group was significantly higher than those in the non-T2DM group (*P* < 0.05). There was no significant difference in BMI or gender between the two groups. Detailed clinical characteristics of the study population were shown in **Table 1**.

**Table 1:** Characteristics of the study subjects.

Variables	T2DM (n = 38)	Non-T2DM (n = 148)	P-value
Age, years	63 ± 10.12	56 (46, 61.75)	< 0.001
Sex, n (%)			0.648
Male	18 (47.37)	64 (43.24)	—
Female	20 (52.63)	84 (56.76)	—
BMI, kg/m^2^	24.80 ± 2.96	23.63 (21.89, 26.12)	0.111
FPG, mmol/L	7.48 (5.79, 10.82)	4.94 (4.69,5.39)	< 0.001
TG, mmol/L	1.51 (1.02, 2.39)	1.07 (0.81,1.57)	0.002
TC, mmol/L	4.67 (4.07, 5.78)	4.68 (4.21,5.48)	0.768
HDL-C, mmol/L	1.24 ± 0.45	1.28 ± 0.40	0.627
LDL-C, mmol/L	2.76 (2.26, 3.49)	2.57 (2.18, 3.07)	0.288
SBP, mmHg	140.00 (120.00, 150.00)	120.00 (110.00, 130.00)	< 0.001
DBP, mmHg	80.00 (71.75, 90.00)	80.00 (70.00, 80.00)	0.012
Mean RSF volume, cm^3^	29.86 ± 12.19	26.45 ± 8.74	0.112
Mean RSF FF, %	34.92 ± 8.21	29.00 ± 7.36	< 0.001
Mean RSF R2^*^, Hz	23.13 ± 4.15	21.62 ± 3.54	0.025

BMI, body mass index; FPG, fasting plasma glucose; TG, triglycerides; TC, total cholesterol; HDL-C, high-density lipoprotein cholesterol; LDL-C, low-density lipoprotein cholesterol; SBP, systolic blood pressure; DBP, diastolic blood pressure; RSF, renal sinus fat; FF, fat fraction.

Data were expressed as mean ± SD, median (25th and 75th percentiles) or n (%); P-value shows comparison of the T2DM and non-T2DM groups.

### 3.2 Consistency analysis

Consistency of the data was shown in **Table 2**. The ICC values were all higher than 0.90, which suggested good intra-observer reproducibility and inter-observer agreement.

**Table 2:** Two-observer measurement consistency.

RSF parameters	Radiologist A1	Radiologist A2	ICC 1*	Radiologist B	ICC 2*
Mean RSF volume, cm^3^	26.92 ± 8.08	24.77 ± 7.80	0.951	24.27 ± 7.96	0.969
Mean RSF FF, %	31.53 ± 8.05	32.51 ± 7.88	0.971	33.27 ± 8.45	0.969
Mean RSF R2*, Hz	22.22 ± 3.48	22.56 ± 3.37	0.981	22.81 ± 3.62	0.980

*ICC 1 is the intra-observer ICC value and ICC 2 is ICC inter-observer value.

### 3.3 Comparison of RSF parameters between the T2DM and non-T2DM groups

**Figure 2:**
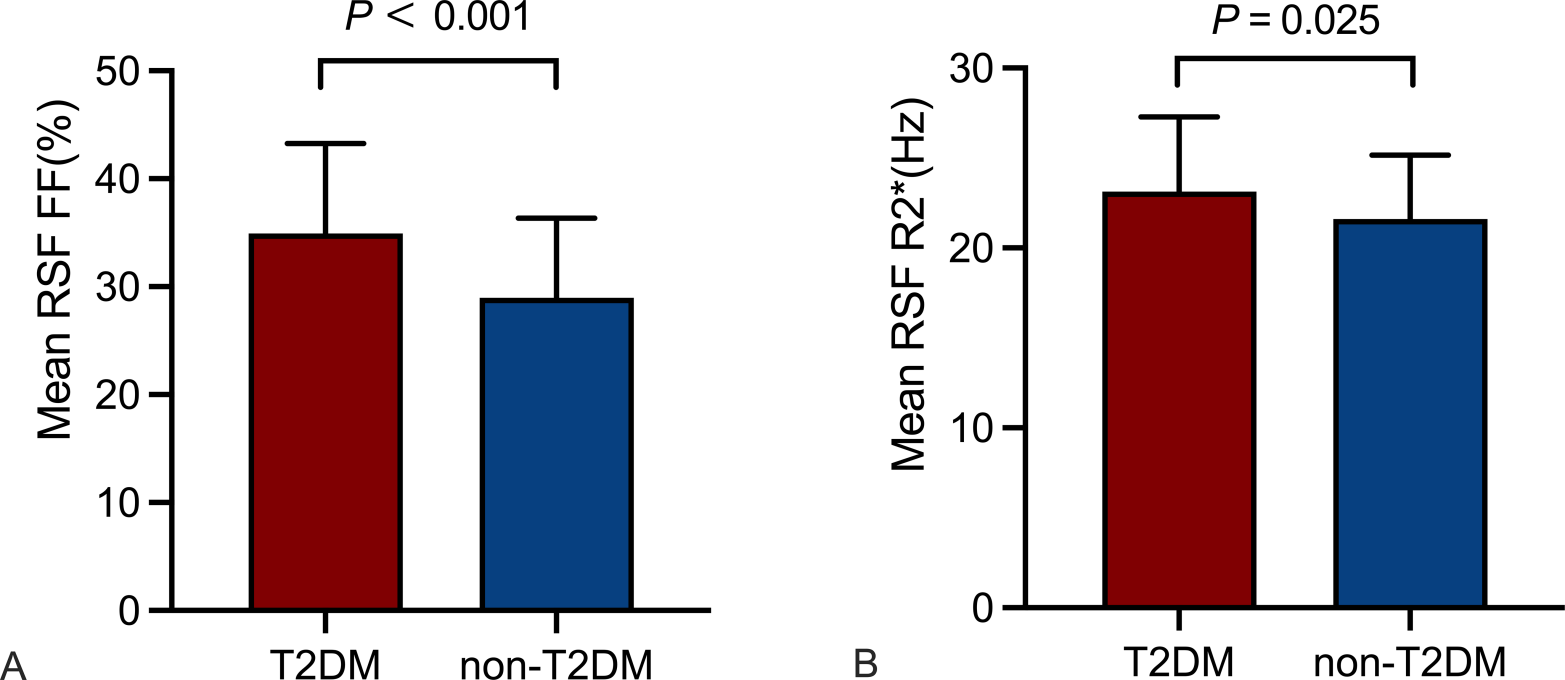
Comparison of RSF parameters between the T2DM and non-T2DM groups. Mean RSF FF **(A)**, mean RSF R2* **(B)** of the T2DM group were higher than those of the non-T2DM group (P < 0.05). RSF, renal sinus fat; FF, fat fraction.

Mean RSF FF and R2* of the T2DM group were 34.92% and 23.13 Hz, which were significantly higher than those of the non-T2DM group (29.00% and 21.62 Hz, *P* < 0.05). It revealed no statistical differences in mean RSF volume between the two groups (**Table 1**, **Figure 2**).

### 3.4 Association between T2DM and parameters of RSF

Univariate logistic regression analyses show the significant correlation of mean RSF FF (OR (95%CI): 1.111 (1.054 - 1.171), P < 0.001) and R2* (OR (95%CI): 1.120 (1.013 - 1.237), P < 0.027) with the presence of T2DM. Multivariate analysis showed that mean RSF FF (OR: 1.231, 95% CI: 1.098 - 1.380) is independently associated with T2DM after adjusting for confounding factors of age, sex and BMI (**Table 3**).

**Table 3:** Association between T2DM and parameters of RSF.

Variables	Univariate analysis		Multivariate analysis	
	P	OR (95%CI)	P	OR (95%CI)
Mean RSF FF	< 0.001	1.111 (1.054 - 1.171)	0.004	1.094 (1.029 - 1.164)
Mean RSF R2*	0.027	1.120 (1.013 - 1.237)	0.446	1.046 (0.931 - 1.175)

RSF, renal sinus fat; FF, fat fraction; OR, odds ratio; CI, confidence interval.

**Figure 3:**
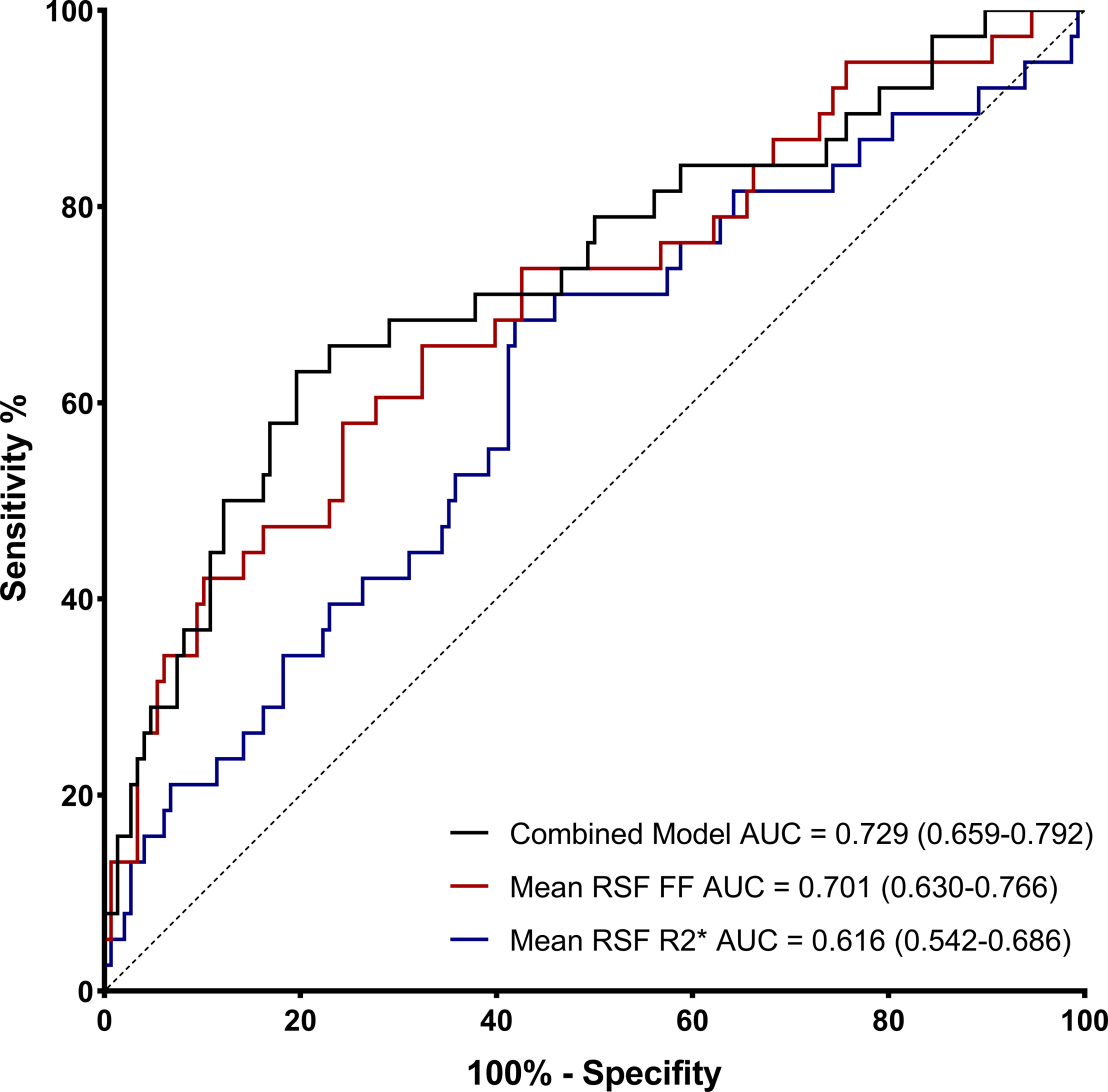
Receiver operating characteristic (ROC) curves of mean RSF FF and R2* for identifying T2DM.

ROC curves evaluated the effect of mean RSF FF and R2* for identifying T2DM. The AUC of mean RSF FF was 0.701 (0.630 - 0.766) with the sensitivity and specificity of 57.89% and 75.68%, respectively, when using 34.40% as the cut-off value. The AUC of mean RSF R2* was 0.616 (0.542 - 0.686) with the sensitivity and specificity of 68.42% and 58.11%, respectively, when using 21.97 Hz as the cut-off value (**Table 4**, **Figure 3**).

**Table 4:** The efficacy analysis of mean RSF FF and R2* for identifying T2DM.

Parameters	AUC (95%CI)	Cut-off value	Sensitivity (%)	Specificity (%)	*P*-value
Mean RSF FF	0.701 (0.630 - 0.766)	34.40	57.89	75.68	< 0.001
Mean RSF R2*	0.616 (0.542 - 0.686)	21.97	68.42	58.11	0.029
Combined model	0.729(0.659 - 0.792)	–	63.16	80.41	< 0.001

AUC, area under the ROC curve; CI, confidence interval; RSF, renal sinus fat; FF, fat fraction.

Furthermore, according to the comparison of AUCs between mean RSF FF and R2*, mean RSF FF presented significantly higher diagnostic efficacy for T2DM than R2* (P < 0.05). After combining the two variables, the performance in identifying T2DM was improved (AUC = 0.729).

### 3.5 Correlations among mean RSF volume, FF and R2*

Mean RSF volume, FF and R2* were correlated with each other after adjusting for age, sex and BMI. Mean RSF volume was significantly positively associated with mean RSF FF (r = 0.384, *P* < 0.001) and mean RSF R2* (r = 0.465, *P* < 0.001). Specifically, there was a higher correlation between mean RSF FF and R2* (r = 0.763, *P* < 0.001).

## 4 Discussion

In this study, it was found that mean RSF FF and R2* were significantly associated with T2DM, and mean RSF FF was the independent risk factor of T2DM. And mean RSF FF presented a higher efficacy than R2* in the identification of T2DM. Moreover, combining mean RSF FF and R2* improved the model performance.

Previous studies showed that visceral adipose tissue and perivascular adipose tissue both play important roles in metabolism, through mechanisms such as endocrinology and immunology (7, 21–23).VAT was found to play a critical role in the development of T2DM (24). Excess free fatty acids in VAT, in conjunction with local inflammation due to cytokines secreted by visceral adipocytes, have been demonstrated to be associated with IR (25). Moreover, findings have indicated that visceral adiposity better characterizes the progression of chronic kidney disease (CKD) (26). Even minor accumulation of adipose tissues in the abdominal region of non-obese metabolic-unhealthy people is associated with a considerably adverse metabolic risk (27, 28). As a kind of ectopic fat, considering its special anatomic localization, RSF has the two identities of perivascular fat and visceral fat simultaneously. Mechanistically, on the one hand, excessive accumulation of RSF can elevate the intra-abdominal pressure, compress the low-pressure renal venous structures and leading to renal volume expansion, increase in renal interstitial pressure, and activation of the renin-angiotensin-aldosterone system (RAAS) (29), which activation may contribute to IR and CKD (30, 31). Moreover, through the mechanical compression of renal vasculature, hypertrophy of RSF leads to inadequate perfusion and hypoxia. This, in turn, triggers cellular stress and the release of pro-inflammatory cytokines, promoting chronic inflammation and renal fibrosis, and ultimately exacerbating the progression of CKD. Concurrently, the interaction of hypoxia and inflammation enhances the activity of the RAAS, which further contributes to hypertension, creating a vicious circle (32–34). On the other hand, due to the similar characteristics with perivascular adipose tissue, as an active endocrine tissue, RSF plays a crucial role in regulating inflammation, vascular function, and metabolism, which may eventually lead to metabolic disorders and CKD (35–37). Since fat content and oxygenation of adipose tissue can be reflected by FF and R2* mapping on MRI respectively (38), it can be assumed that significantly higher mean FF and R2* of RSF in the T2DM group may suggest dysfunctional adipose tissue.

RSF FF, as an index reflecting the quality of RSF, may be related to adipocyte hypertrophy and dysfunction resulting from the excessive lipid depot. The larger the FF in the RSF area, the greater the proportion of lipid components in this tissue area. Moreover, the fat attenuation index (FAI) obtained based on computed tomography (CT) can also reflect tissue fat content. The lower the FAI, the higher the proportion of lipid components in the tissue. In our study, the mean FF value of RSF in the T2DM group was significantly higher than that in the non-T2DM group. We found that the mean RSF FF was an independent risk factor for T2DM after adjusting for confounding factors of age, gender, and BMI, and displayed the better diagnostic performance than RSF R2* for identification of T2DM at a cut-off value of 34.40%. Our study is consistent with the results of the previous study by Lee et al. (10). They found that, when compared with non-T2DM patients, the proportion of lipid components in RSF in T2DM patients is significantly higher by CT-based RS FAI, indicating that the quality of RSF has changed. Furthermore, it suggests hypertrophy of renal sinus adipocytes caused by excessive lipid accumulation (39). Although no *in vitro* study has investigated the association between the mean RSF FF measured on MRI or RS FAI measured on CT and the cellular pathophysiology, it can be assumed that the changes in the qualitative mapping of the adipose tissue may reflect its function (40). Adipocytes have a limited capacity to proliferate and become hypertrophic when storing excessive lipids. As these cells expand, they become less vascularized and hypoxic, leading to adipose tissue dysfunction (3).

The R2* value is obtained by re-aggregating gradients at different times and can non-invasively reflect the RSF hypoxia state. The R2* value is affected by blood flow, tissue oxygenation status adjacent to perfused microvessels, paramagnetic deoxyhemoglobin concentration and ferritin-loaded cells (9, 12). High tissue content of ferritin-loaded cells and deoxygenated hemoglobin yields local magnetic field inhomogeneities producing a phase shift of the hydrogen protons contributing to faster T2* signal decay. So, increase of R2* (1/T2*) suggests iron overload and poor oxygen content in tissue (9, 41). In our study, the R2* value of RSF in T2DM patients was significantly higher than that in the non-T2DM group; yet, it was not the independent risk factor of T2DM. According to the ROC curve of mean RSF R2*, the AUC was 0.616 with the sensitivity of 68.42% and the specificity of 58.11% when identifying T2DM. Previous research has shown increased R2* in adipose tissue indicating the iron content run in parallel to liver iron stores of subjects with obesity (9). It has also been reported that R2* value obtained by IDEAL-IQ correlates with the overexpression of HIF-1α, a classic microvessel density and hypoxic biomarker (12). Adipocyte iron overload and hypoxia in RSF may be the main manifestation of its dysfunction (42, 43). Shi et al. (40) reported high-fat diet feeding in Zucker diabetic fatty rats led to a significant increase in the R2* signal of the perirenal adipose tissue measured by blood oxygen level-dependent MRI (BOLD-MRI). Of note, in their study, R2* was significantly correlated with IR and systemic inflammation. Increased R2* in RSF are in line with recent observations showing increased expression of several iron-related genes in adipose tissue of subjects with obesity and IR (44). High iron levels and low oxygen levels in adipose tissue have multiple relationships and interactive mechanisms, leading to adipose tissue malfunction (45, 46).

In our study, there was no significant difference in the average RSF volume between the T2DM and non-T2DM groups, it corroborates with the results found by Lee et al. (10). They found that there was no significant difference in RSF volume based on CT between groups with and without metabolic syndrome under the non-obese condition. Yet, in the study by Lin et al. (5), T2DM patients demonstrated statistically larger left renal sinus fat volume based on MRI than healthy controls. This may be because there was no significant difference in BMI and gender ratio between the T2DM and non-T2DM groups in our study, but there were higher BMI (30.8 kg/m^2^) and male proportion (50%) in T2DM group in their study. And this can be supported by our previous finding that BMI and gender was associated with RSF volume (17). It also indicates that when metabolic disorder develops in non-obese individuals, change in the proportion of dysfunctional adipose tissue may precede the increase in the volume of RSF. BMI is the most widely used indicator for assessing obesity, yet, a normal BMI may obscure the presence of elevated metabolic risk, since it is ectopic fat deposition rather than BMI that truly reflects metabolic abnormalities (47). The difference in FF and R2* in RSF between T2DM and non-T2DM group, as observed in our study, have the potential to enhance the early detection of this elusive high metabolic risk.

In partial correlation analyses, we focused the relationship between the mean RSF volume, mean RSF FF and mean RSF R2*, after controlling the possible confounders: age, sex and BMI. The mean RSF volume in T2DM patients may correspond to adipocyte hypertrophy in RS. Our study result showed a significant positive correlation between mean RSF volume and mean RSF FF (r = 0.384, *P* < 0.001) and mean RSF R2* (r = 0.465, *P* < 0.001). As mentioned above, hypertrophy caused by excessive accumulation of lipids in adipocytes located in RS can be reflected by FF, and further dysfunction of RSF, manifesting in iron deposition and hypoxia, can be reflected by R2*. We found that there was a significant positive correlation between mean RSF FF and R2* (r = 0.763, *P* < 0.001). This is consistent with the conclusion in previous study that hypertrophy of adipocytes leads to their abnormal function (3). The mean RSF volume, FF and R2* together are indicative of abnormal renal sinus fat function, having potential as imaging markers for early detection of T2DM. Because the field strength of the MRI examination has an impact on the value of R2*, rather than FF (48–51), participants included in our study all underwent MRI examinations with a field strength of 1.5 T using the same type equipment to ensure the accuracy and consistency of the data.

In addition, perirenal adipose tissue is a specific type of visceral adipose tissue located in the retroperitoneal space that has a distinct role in the metabolic system (52), and it can be assessed quantitively by CT, MRI and ultrasound (53–55). Especially, ultrasound. Ultrasound can directly and quickly assess perirenal fat thickness (PrFT) by quantifying the average maximum distance from the posterior part of the kidney to the inner edge of the abdominal wall along the plane of the left and right renal veins (56). Research has demonstrated that the ultrasound-measured PrFT was a contributing independent variable to the estimated 10-year risk of cardiovascular disease and atherosclerotic cardiovascular disease in patients with T2DM (57). Furthermore, it was demonstrated to have a negative and independent correlation with estimated glomerular filtration rate (eGFR), suggesting a potential role for perirenal fat in renal insufficiency in patients with T2DM (58). PrFT based on ultrasound may provide a convenient, cost-effective, and widely available method of assessing metabolic status and renal function. Advancements in ultrasound technology, such as multiparametric ultrasound, three-dimensional ultrasound, contrast-enhanced ultrasound, and super-resolution ultrasound, offer effective methods for the early detection and differentiation of renal disease patterns in the setting of metabolic abnormalities, facilitating therapy adjustment and targeted prevention (59–62). Further research using multimodal approaches, integrating MRI and ultrasound modalities, is anticipated to yield additional insights and refine the understanding of ectopic adipose deposition.

The strength of our study is that we incorporated the highly reproducible qualitative and quantitative measures of RSF by simultaneous non-invasive MRI FF and R2* mapping and investigated the associations between quality and quantity of RSF and T2DM after adjusting age, sex and BMI. However, our study had several limitations. First, this retrospective study could not infer cause and effect, and a longitudinal study would be necessary to investigate the progression of RSF at different stages of T2DM. Second, the RSF was manually segmented, which was time-consuming and subjectively dependent, and the semi-automatic or automatic segmentation methods should be explored in future studies. Third, Since the R2* value can be significantly impacted by the magnetic field strength, participants included in our study all underwent MRI at 1.5 T to ensure the measurement accuracy and consistency. Therefore, the results by the R2* mapping of this study are only expected to be applicable to 1.5 T MRI examinations. Fourth, our study sample was composed of Chinese subjects, so it may limit the generalization of our results to other racial populations. Fifth, the number of enrolled subjects was still relatively small, which was probably why the AUC values in our study was not so high, and further studies with enlarged sample size are expected for stronger confirmation of our findings.

## 5 Conclusion

Simultaneous FF and R2* mapping by MRI can non-invasively reflect the RSF dysfunction in T2DM patients. Our results suggest that the higher FF and R2* of RSF are associated with T2DM, and FF showed the better diagnostic performance for identifying T2DM. These findings indicate the hypertrophy of adipocytes, excessive iron deposition, and hypoxia in RSF, which may represent dysfunction of RSF for T2DM. Furthermore, it suggests that MR-derived RSF FF and R2* could potentially be new markers for image-based measurements of site-specific abdominal fat tissue for early identifying T2DM and guiding personalized T2DM management. It also indicates RSF as a potential therapeutic target for IR andT2DM. In future studies, the combination of both MRI and ultrasound techniques for multimodal data of various RSF parameters will facilitate early detection of T2DM and CKD, as well as help in T2DM patient stratification and efficacy assessment.

## Data Availability

The raw data supporting the conclusions of this article will be made available by the authors, without undue reservation.
